# Advances in Modern Channel Coding

**DOI:** 10.3390/e28010129

**Published:** 2026-01-22

**Authors:** Yongpeng Wu, Peihong Yuan

**Affiliations:** 1 Department of Electronic Engineering, Shanghai Jiao Tong University, Shanghai 200433, China; yongpeng.wu@sjtu.edu.cn; 2 Institute of Space Internet, Fudan University, Shanghai 200433, China

Channel coding has long stood at the core of reliable communications, shaping the evolution of modern information and communication systems. From classical algebraic codes to capacity-approaching schemes such as Turbo codes, low-density parity-check (LDPC) codes, and polar codes, decades of research have continuously pushed the limits of performance, complexity, and practical feasibility. Today, the landscape of channel coding is being reshaped once again by emerging application requirements, ranging from ultra-reliable low-latency communications and short-packet transmission to high-speed optical links, intelligent networks, and data-driven system design.

Recent years have witnessed remarkable progress not only in code construction and decoding algorithms, but also in cross-layer and cross-domain integration. Advances in probabilistic shaping, coded modulation, joint source channel coding, and machine-learning-assisted designs have significantly expanded the traditional boundaries of channel coding theory. At the same time, new challenges remain unresolved, including finite-length performance limits, decoding complexity under stringent latency constraints, robustness to model uncertainty, and adaptability to heterogeneous communication environments.

This Special Issue, Advances in Modern Channel Coding, was conceived to capture these developments and to highlight both theoretical and practical innovations that address the evolving demands of modern communication systems. The five contributions collected in this Special Issue reflect the diversity and vitality of current research in channel coding, spanning fundamental code construction, decoding algorithm optimization, learning-based signal shaping, and system-level integration.

On the theoretical side of code design, Xu et al. investigate LDPC codes constructed from balanced incomplete block designs (BIBDs), providing a rigorous analysis of Tanner graph girth and cycle structures [contributions 1]. By explicitly characterizing short cycles and their multiplicities, this work offers valuable design insights for structured LDPC codes with predictable graph properties and strong error-correction performance, contributing to the broader effort of analytically grounded LDPC code construction.

Polar codes, as a cornerstone of modern channel coding and a key component of 5G systems, are the focus of several contributions in this Special Issue. Xu et al. address the finite-length limitations of non-binary polar codes by proposing a bit-level construction framework for multiplicative-repetition-based designs [contributions 2]. By allowing synthesized channels to carry a variable number of information bits and combining analytical bounds with Monte-Carlo-based optimization, this work significantly improves decoding performance and enables more flexible utilization of non-binary polarization. From the decoding perspective, Pillet et al. propose restart mechanisms for successive-cancellation list-flip (SCLF) decoding of polar codes [contributions 3]. Their work tackles the practical challenge of variable decoding latency, demonstrating that substantial reductions in average execution time can be achieved with only marginal memory overhead.

Beyond classical code construction and decoding, this Special Issue also highlights emerging paradigms that integrate channel coding with learning-based optimization and system-level design. Ji et al. introduce a mutual-information neural-estimation-driven framework for constellation shaping, enabling geometric, probabilistic, and joint shaping without explicit channel state information [contributions 4]. This work exemplifies how deep learning can be judiciously employed to optimize coded modulation schemes, striking a favorable balance between performance gains and implementation complexity. At the system level, Li et al. explore the joint design of polar codes and physical-layer network coding for relay-assisted visible light communication systems [contributions 5]. Their results demonstrate that coding-aware network design can simultaneously improve reliability, throughput, and coverage, highlighting the growing importance of cross-layer optimization in emerging communication scenarios.

Collectively, these contributions address several important gaps in the current literature: the need for structured yet flexible code constructions, decoding algorithms that balance performance with latency and complexity, and adaptive coding and modulation strategies capable of operating under imperfect channel knowledge and diverse system constraints. The Special Issue thus provides a representative snapshot of how modern channel coding research is evolving from isolated component design toward holistic, intelligent, and application-driven solutions.

Looking ahead, the future of channel coding research is rich with opportunities and challenges. Promising directions include ultra-short block-length coding and decoding, joint optimization of coding, modulation, and detection under stringent latency and energy constraints, and robust designs for highly dynamic and heterogeneous networks such as satellite constellations, integrated terrestrial–non-terrestrial systems, and optical wireless communications. Moreover, the principled fusion of information-theoretic foundations with machine learning techniques—ensuring reliability, interpretability, and generalization—remains an open and compelling research frontier. Finally, as communication systems increasingly interact with sensing, computing, and networking functionalities, channel coding will continue to play a central role in enabling reliable and efficient information exchange.

We hope that this Special Issue will not only serve as a valuable reference for researchers and practitioners, but also stimulate further exploration and innovation in modern channel coding. We sincerely thank all authors for their high-quality contributions and the reviewers for their dedicated efforts. We also express our gratitude to the editorial team for their support throughout the process. It is our belief that the ideas presented in this Special Issue will inspire continued progress in this vibrant and essential field.

